# Deep Brain Stimulation for Obesity: A Review and Future Directions

**DOI:** 10.3389/fnins.2019.00323

**Published:** 2019-04-18

**Authors:** Douglas A. Formolo, Joana M. Gaspar, Hiago M. Melo, Tuany Eichwald, Ramiro Javier Zepeda, Alexandra Latini, Michael S. Okun, Roger Walz

**Affiliations:** ^1^Center for Applied Neuroscience, University Hospital, Federal University of Santa Catarina, Florianópolis, Brazil; ^2^Graduate Program in Neuroscience, Federal University of Santa Catarina, Florianópolis, Brazil; ^3^Laboratory of Bioenergetics and Oxidative Stress, Department of Biochemistry, Federal University of Santa Catarina, Florianópolis, Brazil; ^4^Graduate Program in Biochemistry, Department of Biochemistry, Federal University of Santa Catarina, Florianópolis, Brazil; ^5^Department of Neuroscience, Faculty of Medicine, Chile University and Health Science Institute, O’Higgins University, Santiago, Chile; ^6^Fixel Institute for Neurological Diseases, Department of Neurology, University of Florida, Gainesville, FL, United States; ^7^Graduate Program in Medical Sciences, Federal University of Santa Catarina, Florianópolis, Brazil; ^8^Department of Internal Medicine, University Hospital, Federal University of Santa Catarina, Florianópolis, Brazil

**Keywords:** obesity, deep brain stimulation, hypothalamus, nucleus accumbens, metabolic disorders, neuroinflammation

## Abstract

The global prevalence of obesity has been steadily increasing. Although pharmacotherapy and bariatric surgeries can be useful adjuvants in the treatment of morbid obesity, they may lose long-term effectiveness. Obesity result largely from unbalanced energy homeostasis. Palatable and densely caloric foods may affect the brain overlapped circuits involved with homeostatic hypothalamus and hedonic feeding. Deep brain stimulation (DBS) consists of delivering electrical impulses to specific brain targets to modulate a disturbed neuronal network. In selected patients, DBS has been shown to be safe and effective for movement disorders. We review all the cases reports and series of patients treated with DBS for obesity using a PubMed search and will address the following obesity-related issues: (i) the hypothalamic regulation of homeostatic feeding; (ii) the reward mesolimbic circuit and hedonic feeding; (iii) basic concepts of DBS as well as the rationale for obesity treatment; (iv) perspectives and challenges in obesity DBS. The small number of cases provides preliminary evidence for the safety and the tolerability of a potential DBS approach. The ventromedial (*n* = 2) and lateral (*n* = 8) hypothalamic nuclei targets have shown mixed and disappointing outcomes. Although nucleus accumbens (*n* = 7) targets were more encouraging for the outcomes of body weight reduction and behavioral control for eating, there was one suicide reported after 27 months of follow-up. The authors did not attribute the suicide to DBS therapy. The identification of optimal brain targets, appropriate programming strategies and the development of novel technologies will be important as next steps to move DBS closer to a clinical application. The identification of electrical control signals may provide an opportunity for closed-loop adaptive DBS systems to address obesity. Metabolic and hormonal sensors such as glycemic levels, leptin, and ghrelin levels are candidate control signals for DBS. Focused excitation or alternatively inhibition of regions of the hypothalamus may provide better outcomes compared to non-selective DBS. Utilization of the NA delta oscillation or other physiological markers from one or multiple regions in obesity-related brain network is a promising approach. Experienced multidisciplinary team will be critical to improve the risk-benefit ratio for this approach.

## Introduction

According to the World Health Organization (WHO), obesity is defined as an abnormal and excessive fat accumulation. The increase in fat mass is a major health problem and is a risk factor for the development of several metabolic complications. Obesity decreases the quality of life and decreases life expectancy. The incidence of obesity has continued to rise rapidly during the last several decades. Similarly, the prevalence of obesity has been rising in most countries worldwide, and its prevalence has doubled in more than seventy countries over the last four decades. It was estimated that in 2015 there were a total of 603.7 million obese adults (body mass index [BMI] > 30 kg/m^2^) and 107.7 million obese children (defined as a BMI at or above the 95th percentile). Approximately 1.9 billion adults are by definition overweight (BMI > 25 kg/m^2^) (Gonzalez-Muniesa et al., 2017). The rising prevalence of obesity has been associated with a higher incidence of chronic metabolic disorders including type 2 diabetes. Both diseases share similar features including early cognitive dysfunction and a substantial socio-economic impact (Gaspar et al., 2016; Remor et al., 2018).

The etiology of obesity results from a complex interaction of several factors, including socioeconomic status, genetics, epigenetics, cultural features, and lifestyle. The obesity problem is even more complex with a link to compulsive eating behaviors and to depression (Val-Laillet et al., 2015; Ivezaj et al., 2016). Although no cause-and-effect relationship has been conclusively established, a systematic review revealed that obesity and depression had a significant and bidirectional association. The association with anxiety was modest but potentially important (Rajan and Menon, 2017). Despite its multifactorial etiology, a general consensus has emerged that the majority of obesity cases result from the combination of an increase in food intake along with a decrease in energy expenditure which together results in excessive fat accumulation. In most cases, obesity is a preventable disease and is largely dependent on lifestyle. Living a healthy lifestyle has recently become more of a challenge with elevated levels of stress, reduction of physical activity and the availability of palatable foods that are high in sugar, fat, and calories. The increased availability of an energy-dense diet, high in saturated fat and sugar, has been strongly associated with weight gain and increased adiposity, precipitating a strong change in energy balance not only resulting from a simple increase in energy intake (McClelland et al., 2013).

The palatable and densely caloric foods affect brain circuits involved with the control of energy metabolism in the hypothalamus and those involved with reward and humor perception within the limbic system. In predisposed, individuals this combination may result in overeating patterns and manifest as a “food addiction” or “food abuse.” This phenomenon can increase the difficulty in achieving a long-term successful reduction of caloric intake by non-pharmacological and pharmacological approaches. This phenomenon has also increased the challenges of bariatric surgery for individuals with morbid obesity (Stice et al., 2013). Additionally, genetic factors might contribute to vulnerability to weight gain that, in combination with the environment precipitants result in brain network changes. This may reinforce the desire for food intake even if this desire is incongruent with the aspiration to reduce weight (Horstmann, 2017). Neuromodulation for the regulation of homeostatic and hedonic feeding has been suggested as a potential approach.

We review all the cases reports and series of patients treated with DBS for obesity using a PubMed search and will address the following obesity-related issues: (i) the hypothalamic regulation of homeostatic feeding; (ii) the reward mesolimbic circuit and hedonic feeding; (iii) basic concepts of DBS as well as the rationale for obesity treatment; (iv) perspectives and challenges in obesity DBS.

## Hypothalamus, Homeostatic Feeding, and Obesity

Homeostatic feeding is necessary for basic metabolic processes and survival (Rossi and Stuber, 2018). Energy homeostasis which is defined as a balance between food intake and energy expenditure is regulated primarily by the hypothalamus. The hypothalamus regulates food intake and energy expenditure but also regulates peripheral glucose and lipid homeostasis as well as metabolism (Morton et al., 2014; Timper and Bruning, 2017).

The discovery of the hypothalamus as a key center for energy homeostasis had its genesis with the studies of Hetherington and Ranson (1940) which revealed that adiposity increased in rats with hypothalamic lesions. Additionally, the discovery of leptin in 1996, by Zhang et al. (1994) opened new pathways for the molecular understanding of the role of the hypothalamus in the regulation of food intake and energy expenditure.

**FIGURE 1:**
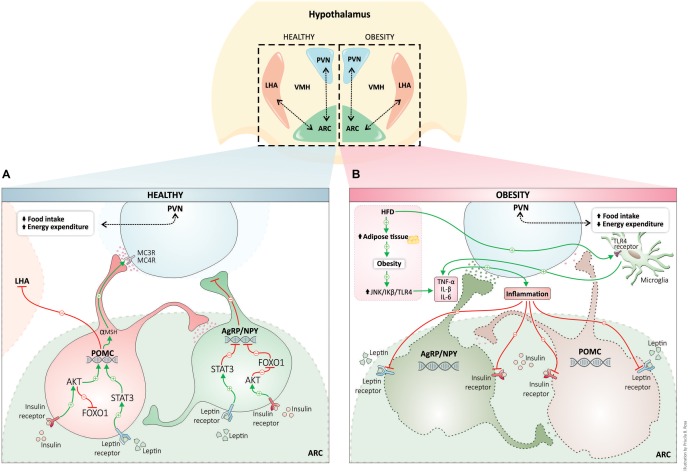
Hypothalamic regulation of energy homeostasis in healthy (blue) and obesity (pink). The neuronal populations in the arcuate nucleus of the hypothalamus, POMC, and AgRP, senses and integrates peripheral adipostatic hormones (insulin and leptin), that circulate in levels proportionate to nutritional status and adipose tissue stores. Under the physiological postprandial state, insulin and leptin bind to respective receptors, both in POMC and AgRP neurons regulating the transcription of neuropeptides. Leptin and insulin signaling pathway stimulates POMC peptide transcription (activation of POMC neurons) and inhibits the transcription of AgRP neuropeptide (leading to AgRP neuronal inhibition). POMC neuronal activation involves the processing of POMC with the formation of α-MSH that is an agonist of MC4R in PVN neurons. PVN activation culminates in satiety (decreased food intake) and stimulation of energy expenditure **(A)**. High-fat diet consumption and obesity induces a whole-body chronic inflammatory state. Proinflammatory cytokines produced during inflammation are responsible for hypothalamic insulin and leptin resistance, and consequently, the neuronal control of energy homeostasis is disrupted inducing an increase in food intake and a decrease in energy homeostasis **(B)**. AgRP, agouti-related protein; Akt, protein kinase B; ARC, arcuate nucleus; FOXO1, forkhead box protein O1; IL-1β, interleukin-1β; IL-6, interleukin-6; JNK, c-Jun N-terminal kinases; LHA, lateral hypothalamus; MC4R, melanocortin 4 receptor; NPY, neuropeptide Y; POMC, proopiomelanocortin; α-MSH, alpha-melanocyte-stimulating hormone; PVN, paraventricular nucleus; STAT3, Signal transducer and activator of transcription 3; TLR4, toll-like receptor 4; TNF-α, tumor necrosis factor-α; VMH, ventromedial hypothalamus.

The Figure 1 shows the interconnected hypothalamic nuclei, including the arcuate nucleus (ARC), the paraventricular nucleus (PVN), the ventromedial nucleus (VMN), the dorsomedial nucleus, and the lateral hypothalamic area (LHA) in both the healthy and the obesity state. Due to its privileged anatomical location, ARC is the principal region to receive and to sense afferent signals (nutrients and hormones) from the gut and the brainstem, as well as the region that processes efferent signals capable of modulating both food intake and energy expenditure. Two well-defined neuronal populations within the ARC have been described and each has been characterized by the expression of specific neuropeptides. These neuronal populations have potent effects on energy homeostasis namely, proopiomelanocortin (POMC) and agouti-related protein/neuropeptide Y (AgRP/NPY) (Morton et al., 2014; Sohn, 2015; Timper and Bruning, 2017). However, more recently it was identified that there was a third neuronal population which expressed tyrosine hydroxylase and had orexigenic characteristics (Zhang and van den Pol, 2016). All of these neuronal populations express high levels of hormone receptors (e.g., insulin, leptin, ghrelin, GLP-1, among others) and these receptors seem to facilitate a response to metabolic signals and to reflect the energy state of the organism as well as to control energy homeostasis (Figure 1A).

The anorexigenic neurons, the POMC, are activated by adiposity signals, such as leptin, insulin, and some nutrients. The binding of leptin to its receptor induces the phosphorylation of the transcription factor STAT3 and this reaction upregulates POMC expression (Hakansson and Meister, 1998; Lee et al., 2018). Neuronal insulin signaling induces phosphorylation of forkhead box protein O1 (FOXO1), which is also a transcription factor that increases the expression of POMC (Belgardt et al., 2008). POMC is a neuropeptide that is post-translationally processed in several active peptides, including α and β melanocyte-stimulating hormone (MSH) and β-endorphins (Liotta et al., 1980; Wardlaw, 2011). After activation, neuronal projections of POMC neurons activate second-order neurons in the PVN, acting on the melanocortin receptors (MC3R/MC4R) leading to a decrease in food intake and to an activation of energy expenditure. The activation of POMC neurons has been shown to decrease body weight (Cheung et al., 1997; Wardlaw, 2011; Mountjoy, 2015). By contrast, the second population of AgRP/NPY neurons, inhibited by leptin and activated by fasting and ghrelin, has been shown to have a potent orexigenic effect, increasing food intake and decreasing energy expenditure (Wu et al., 2014). Ghrelin is primarily produced by stomach cells under fasting conditions. Ghrelin modulates feeding behavior and metabolism through the ghrelin receptor. The ghrelin receptor is abundantly expressed in the hypothalamus, mainly in AgRP/NPY neurons. AgRPq/NPY neurons are depolarized and activated by ghrelin. Concomitantly, ghrelin hyperpolarizes and inhibits POMC neurons (Kamegai et al., 2000; Al Massadi et al., 2017). In the fed state, AgRP expression is suppressed by leptin. Under fasting conditions (no leptin release) there is an increase in AgRP and consequent increases in food behavior and adiposity. Activation of these neuronal populations leads to a release of AgRP, which is an antagonist of the MC4 receptor, and it inhibits PVN neurons. This process blocks the satiety feeling and stimulates feeding behavior through activation of lateral hypothalamic neurons (LHA) (Figure 1A) (Hahn et al., 1998; Luquet et al., 2005; Sohn, 2015).

Obesity is considered by many experts to be a low-grade chronic inflammatory condition. For several years, it was believed that the link between peripheral insulin resistance and obesity was a consequence of several variable factors including feeding patterns, consumption of high-fat diets, sedentary lifestyle and also that obesity occurred from stress. All of these factors are involved in the induction of systemic inflammation which can result in changes in metabolic and endocrine signaling (Hotamisligil, 2017). The expansion of white adipose tissue which occurs during the development of obesity produces proinflammatory cytokines (TNF-α, IL-1β, and IL-6) through the activation of intracellular serine kinases, such as JNK and IkB kinase. These kinases participate in the induction of insulin resistance (see review, Hotamisligil, 2017). Inflammation is also a hallmark in the brain of rodents under peripheral metabolic dysregulation (Remor et al., 2018), and in the hypothalamus of diet-induced obesity models (De Souza et al., 2005; Wang et al., 2012) (Figure 1B). Besides neuroinflammation, hypothalamic dysregulation induced by high-fat diets can result in an increase in markers of oxidative stress, endoplasmic reticulum stress, autophagy defects and changes in the rate of apoptosis and neuronal regeneration (Cavadas et al., 2016).

Unlike inflammation in peripheral tissues, which develops as a consequence of obesity, hypothalamic inflammatory signaling, microglia activation, and gliosis is observed during the first week of high fat diet consumption. They can also be observed prior to weight gain and in ARC (Thaler et al., 2012). A growing body of studies has demonstrated that saturated fatty acids are the trigger for early hypothalamic inflammation and are mediated mainly by microglial cells and astrocytes (Kleinridders et al., 2009; Gupta et al., 2012; Valdearcos et al., 2014, 2017; Mendes et al., 2018). Toll-like receptor 4 (TLR4) can be activated by saturated fatty acids, inducing the activity of NF-κB which transactivates gene expression of various proinflammatory cytokines and oxidative stress (Zhang et al., 2008; Kleinridders et al., 2009; Milanski et al., 2009). NF-κB also activates the expression of the suppressor of cytokine signaling 3 (SOCS3) which promotes negative feedback in the insulin and leptin intracellular signaling pathways, potentially linking hypothalamic inflammation with central leptin and insulin resistance (Valdearcos et al., 2015). Blocking the inflammatory process in the hypothalamus can prevent diet-induced obesity and insulin/leptin resistance (Kleinridders et al., 2009; Valdearcos et al., 2014, 2017; Mendes et al., 2018) (Figure 1B).

Neuroinflammatory damage to ARC neurons might disrupt integrated circuitry, including the communication with the downstream effector nucleus (such as PVN and LHN), and may generate a pathologic activation pattern that disturbs energy homeostasis. Targeting hypothalamic structures with DBS could thus possibly lead to weight loss and reduction in binge eating behavior if the appropriate circuits can be selectively activated or inactivated (Whiting et al., 2013).

## Reward Circuits, Hedonic Feeding, and Obesity

The reward circuit structures have been implicated in motivation and desire, associative learning, and in emotions with a pleasure component related to reward. ‘Liking’ mechanisms include hedonic circuits that connect forebrain limbic structures such as NA and ventral pallidum (where opioid/endocannabinoid/orexin signals can amplify sensory pleasure). ‘Wanting’ mechanisms include larger opioid networks in NA, striatum, and amygdala that extend beyond the hedonic hotspots, as well as mesolimbic dopamine systems, and corticolimbic glutamate signals that interact with those systems (Berridge et al., 2010). Hedonic feeding is driven by sensory perception or pleasure involving reward structures and these also show a close anatomic and functional relationship with the hypothalamus and the homeostatic feeding (Rossi and Stuber, 2018). Hyperpalatable foods may be potentially “addictive” and have been compared to drugs of abuse. The cues that predict drug and food reward activate similar regions that have been implicated in reward and reward learning. The circuits involved include the mesolimbic dopamine system, which projects from the ventral tegmental area (VTA) to the NA (Stice et al., 2008). Functional MRI has shown that blood oxygen dependent signal (BOLD) in the NA is selectively increased during the perception of pleasant, emotionally arousing pictures and during mental imagery of pleasant, emotional scenes (Sabatinelli et al., 2007; Costa et al., 2010). In comparison to lean individuals, obese individuals experience greater activation in the gustatory cortex and somatosensory regions in response to anticipation and consumption of food (consummatory and anticipatory food reward) and experience a weaker activation in the striatum during food intake (Stice et al., 2008). The findings of reward responsivity in obese people can be inherited (genetic predisposition) or can result from repeated food intake or from both scenarios (Berridge et al., 2010).

On the other hand, the incidental finding of increased weight gain after discontinuation of pharmacological dopaminergic in rats suggested that hypofunctioning of dopamine circuits related to overstimulation by high palatable function could contribute to obesity (Reinholz et al., 2008). The Taq1A minor (A1) allele of the gene codifying dopamine receptors 2 and 3 (DRD2/3 gene) is associated with lower DRD2/3 density and has been found to exist in higher frequencies in obese subjects (Dang et al., 2016). In rodents, reduced DA transmission has been well documented in the striatum of obese rats (Johnson and Kenny, 2010; Rada et al., 2010). The results were in agreement with the earlier report showing lower striatal levels of D2 receptors of obese subjects in comparison to controls in 2001 (Wang et al., 2001). However, a large sample of adult subjects (*n* = 130, age between 18 and 81 years), Dang et al. (2016) show no association between o relation between DRD2/3 and BMI (range from underweight to extreme obesity) after controlling for age distribution. Besides that, a randomized pilot study with a dopamine agonist (cabergoline) treatment for 16 weeks did not affect significantly the weight loss, but improve the glucose tolerance (Gibson et al., 2012). Also, genetic studies in humans have suggested that striatal D2Rs for obesity etiology have been considered somewhat controversial. Experts have questioned the reward deficiency theory of “food addiction” (Benton and Young, 2016). Recently Labouesse et al. (2018) outlined a causal relationship between striatal D2Rs and obesity in mice. These authors showed that high striatal D2R during development increased the risk for obesity in the mouse and that obesogenic diets were necessary to reveal the full effects of the D2R on obesity in the mouse. This data suggested that diet was an important cofactor along with the genetic predisposition.

Although the genetic and neuroimaging studies in humans and the experimental findings in rodents suggest a complex role for the reward-related dopaminergic system in obesity, the results support the use of this circuit as a target for “stimulation” or “inhibition” by DBS.

## Deep Brain Stimulation

Based partially on the notion that high-frequency stimulation could suppress extrapyramidal tremor during functional ablative neurosurgery (Hassler et al., 1960; Alberts et al., 1965, 1966), Benabid et al. (1987) moved the field toward chronic stimulation with high-frequency continuous stimulation (130 Hz) of the thalamic nucleus ventralis intermedius for essential tremor and for parkinsonian tremor. He showed that DBS could be applied bilaterally without pseudobulbar and cognitive side-effects, an important observation as this was the major shortcoming of bilateral lesion therapy. Collectively, these observations consolidated the concept that high-frequency DBS could provide a “functional” lesion or alternatively modulation of a relevant brain network (Grill et al., 2004). We do not completely understand the mechanism of action of DBS, however, there seem to be important associated neurophysiological, neurochemical, neurovascular, neurogenic, neurochemical (e.g., glial cells), and neuro-oscillatory changes. The application of DBS should always be focused to selectively influence a specific and even multiple brain targets to modulate a relevant neural network. It should only be applied to alleviate human suffering (Benabid et al., 1987; Miocinovic et al., 2013; Williams and Okun, 2013; Okun, 2014).

The target size and the anatomical relationship to adjacent structures should be carefully considered during surgical planning. Presently, most DBS leads contain four platinum/iridium cone shape electrodes around the lead with a 1.27 mm diameter and 1.5 mm length. The spacing between each electrode can be 1.5 mm or 0.5 mm providing, respectively, a total of 10.5 mm or 7.5 mm length distance between the deepest and the most superficial electrode borders. There are newer models with eight contacts and segmented leads with variable spacing. The DBS leads are inserted by stereotactic surgery into the targeted brain structure, and an extension wire is subcutaneously tunneled to connect the implanted pulse generator (IPG) to the DBS lead (Oluigbo et al., 2012; Okun, 2014; Klooster et al., 2016). The IPG delivers adjustable pulses through the quadripolar electrodes and can be programmed to many settings based on voltage (or current in newer devices) amplitude, pulse width, and frequency (Hassler et al., 1960; Alberts et al., 1966; Benabid et al., 1991; Kuncel and Grill, 2004; Kringelbach et al., 2007; Brocker and Grill, 2013; Udupa and Chen, 2015). The DBS can be switched ON or OFF on demand (Oluigbo et al., 2012) in some paradigms. When the monopolar configuration is utilized a large sphere of current is generated and when bipolar is used the current is more restricted and elliptical. Newer devices may provide multiple independent current sources as well as segmented leads which can be used to steer or shape the current.

Deep brain stimulation has steadily become a safer procedure especially when an experienced multidisciplinary approach is applied. Severe adverse events are usually less than 5% in well-selected cases (Shukla et al., 2015). DBS is FDA approved in the United States for the treatment of advanced Parkinson’s disease (Odekerken et al., 2013), dystonia (Meoni et al., 2017), and essential tremor (Barbe et al., 2018) and also has a humanitarian device exemption for dystonia and obsessive-compulsive disorder. DBS has been utilized in several refractory neuropsychiatric disorders including Tourette syndrome, depression (Schlaepfer et al., 2013), post-traumatic stress disorder (Koek et al., 2014), epilepsy (Salanova, 2018), obsessive-compulsive disorder (Goodman et al., 2010), pain (Farrell et al., 2018), Alzheimer’s disease (Leoutsakos et al., 2018); drug addiction (Wang et al., 2018) and appetite disorders (Whiting et al., 2018).

Several observations have described a “feeling of happiness” accompanied by smile that progressed to natural laughter with DBS applied to the internal capsule/NA region for obsessive-compulsive disorder (Okun et al., 2004, 2007; Springer et al., 2006; Haq et al., 2011). Mood elevation during stimulation-induced smiles and laughter could be transient and was found to habituate with chronic NA stimulation (Springer et al., 2006; Haq et al., 2011). Rarely DBS induced mood elevation or “manic-like” symptoms in PD which were rapidly reversed with IPG reprogramming. Also, non-mood-related symptoms may also occur. Responses such as taste, smell, and smile were strongly associated with the most ventral lead positions. Similarly, physiological responses – for example, autonomic changes, increased breathing rate, sweating, nausea, cold sensation, heat sensation, fear, panic and panic episodes were significantly associated with ventral stimulation (Okun et al., 2007). Some authors showed that resistant depression treated with medial forebrain bundle DBS may improve over weeks to months (Schlaepfer et al., 2013; Fenoy et al., 2016). Perhaps most compelling was a paper by Halpern et al. (2011) which showed a delta oscillation in NA that was associated with feeding behavior in the mouse. These investigators also explored the use of neuromodulation applied to the NA in the animal and in one human case (an OCD case) as a method to modulate this “feeding” oscillation.

Taken together, clinical observations have shown that modulation can affect different limbic symptoms and this response can be exhibit variable latency. Neuromodulation for obesity treatment will need to consider the overlapping circuits involved with hedonic and homeostatic feeding. Selectivity of a DBS treatment to specific cells using optogenetics may also be a viable approach (Creed et al., 2015; Lüscher et al., 2015; Mastro et al., 2017).

## Hypothalamic DBS as Applied to Obesity

The hypothalamic nuclei have become targets for DBS therapy in obesity because of the known association with vegetative functions, social behavior, food behavior and control of metabolism. Human hypothalamus is a 0.7 cm^3^ volume and it is functionally heterogeneous and complex. It is located below the thalamus and it forms the lateral walls and floor of the third ventricle (Saper, 2004). The four hypothalamic nuclei directly involved with appetite control and food behavior are the VMH, LHA, DMH, PVN, and ARC (Lemaire et al., 2013; Barbosa et al., 2017). These are the nuclei of interest for neuromodulation.

Classical ablative studies have established VMN as the “satiety center,” and LHA as the “feeding center.” This dichotomy has, however, been replaced by a more complex hypothalamic neural network coupled to hormonal, metabolic and energy regulation signals (Lacan et al., 2008) as shown in Figure 1. In both rodent models and in humans obesity has been associated with hypothalamic gliosis and neuronal injury and this has been raised by some experts as a concern for future neuromodulation therapy (Thaler et al., 2012). Variations in the pattern of gliosis and also of neuronal injury distribution may impact local and distal neuroplasticity and may have implications for clinical outcome. The two nuclei require different stimulation frequencies to produce their therapeutic effects (Hamani et al., 2008; Wilent et al., 2010). Moreover, as previously described, systemic metabolic signals under fasting or non-fasting conditions (such as leptin and ghrelin) have antagonistic effects over ARC neurons (namely the POMC and AgRP) and result in the selective activation of PVN or LHA, which either favors weight loss or stimulates food behavior (Morton et al., 2014; Timper and Bruning, 2017).

As shown in Figure 1, in healthy conditions the PVN activation culminates in satiety (decreased food intake) and stimulation of energy expenditure. This nucleus is modulated by the anorexigenic POMC and orexigenic AgRP/NPY neurons from ARC nucleus. It has been suggested that to be effective for obesity, the PVN should be activated by a device directly implanted into the nucleus itself or in the ARC nucleus that projects to the PVN. A DBS neuromodulation approach of the ARC to treat obesity would preferably activate selectively the anorexigenic POMC and/or inactivate the orexigenic AgRP/NPY neurons. Because obesity is associated with complex changes in the neurophysiology of the POMC and AgRP/NPY neurons (see Figure 1), the result of ARC neuromodulation by DBS on the PVN in obese patients is tricky and an optogenetic manipulation may be a preferred approach. Also, because the PVN and ARC nuclei are relatively small, their selective modulation by DBS remains a barrier.

The DMH has been implicated in day-time feeding, in emotional responses to stress and when lesioned in male and female patients has been shown to be associated with persistent impotence and loss of libido (Meyers, 1962; Barbosa et al., 2017). Implications for DMH as a target for DBS in obesity remain to be explored.

The series and case reports about the VMH, LHA, and NA as DBS targets for obesity are discussed below and also summarized in Table 1.

**Table 1:** Series and cases reports of patients treated with DBS for obesity.

References	Target	Stimulations parameters	*N*	Efficacy assessments	Follow-up (months)	Main results	Side effects
Hamani et al., 2008	Ventromedial hypothalamus (bilateral)	50 Hz, 210 μs and 3–4 V	1 (♂)	Body weight	5 months	↓ Body weight	Warming sensation (>4 V), flashes of light
				Neuropsychological assessment		↑ Memory	
				Binge eating			
Wilent et al., 2010	Ventral medial hypothalamus (bilateral)	135 Hz, 60 μs and 1–7 V	1 (♀)	–	–	–	Panic attack (increase in heart rate and blood pressure and anxiety feelings)
Whiting et al., 2013	Lateral hypothalamic (bilateral)	185 Hz, 90 μs and 1–7 V	3 (2♀; 1♂)	Body weight	35 months		Nausea, anxiety or “hot of flushed” sensations during programming changes
				Psychological testing		↓ Body weight (2/3)	
						↓ Body weight (2/3)	
				Binge Eating Scale		↓ Binge eating behaviors (1/3)	
				Body shape		No changes in Psychological Scores and life quality (3/3)	
				Quality of life		↑ Body image	
Talakoub et al., 2017	Lateral hypothalamic (bilateral)	8 Hz, 90 μs, and 3 V	1 (♂) a Prader– Willi	Local field potentials (LFPs) from the DBS contacts during acute evaluation during hunger and satiety.		Exposure to food-related cues during hunger induced an increase in beta/low-gamma activity. Satiety was marked by alpha rhythms (8 Hz). Alpha frequency DBS delivered prior to and during food intake resulted in sensation of fullness without effects on crave for food. Body weight was not reported.	None
Franco et al., 2018	Lateral hypothalamic (bilateral)	Off (2 months)	4 (2♀; 2♂) all were Prader– Willi	Calorimetry, bioimpedanciometry, neuropsychological assessments, hormonal levels, blood workup, and sleep studies	6 months	No major effects on anthropometric and calorimetric variables, hormonal levels, blood workup, sleep and neuropsychological evaluation	Two had stimulation-induced manic symptoms, one improves with discontinuation of DBS and one required topiramate increase.
		40 Hz (1 month)					One removes the system because infection and one successfully treated with antibiotics
		15 days washout					
		130 Hz (1 month)					
Mantione et al., 2010	Nucleus accumbens (bilateral)	185 Hz, 90 μs and 3.5 V	1 (♀)	Psychological assessment (anxiety and depression)	24 months	↓Obsessive–compulsive symptoms	None reported
				Body weight		↓Body weight	
						↓ Anxiety	
						↓ Depression	
Harat et al., 2016	Nucleus accumbens (bilateral)	130 Hz, 208 μs and 2–3, 75 mA	1 (♀)	Body weight, body mass index, and neuropsychological assessment	14 months	↓ Weight	None
						↓ BMI	
						Without neuropsychological impairment	
Tronnier et al., 2018	Nucleus accumbens (bilateral)	130 Hz, 90 μs and 3–4 V	1 (♀)	Body weight, Binge Eating Scale, Weight Efficacy Life-Style, Depression Scales, SF36 mood, simples mood	14 months	↓ Body weight	Difficulties to falling sleep
						↓ Resist desire to eat and binge eating behaviors	
						↓ Depressed symptoms	
						↑ Mood and quality of life	
Rezai et al., 2018	Nucleus accumbens (bilateral)	Not reported (high-frequency)	3 (♀)	Body weight, body mass index	36 months	One patient finished the follow-up period with ↓ weight and ↓ BMI	One patient withdrew from the study with 13 months; one patient completed suicide after 27 months in the study, non-attributed to DBS (see the main text)
						Two patients did not complete the trial	


### Ventral Medial Hypothalamus DBS

Hamani et al. (2008) performed the first DBS trial to treat human obesity. The trial was inspired by the positive results obtained from preclinical (Halpern et al., 2011; Prinz and Stengel, 2018) and clinical studies, and it revealed that inhibition of the hypothalamus resulted in decreased food intake and weight loss (De Salles et al., 2018). Hamani et al. (2008) implanted DBS leads bilaterally in the ventral hypothalamus of one 50-year-old man with a life-long history of obesity (190.5 kg; BMI, 55.1 kg/m^2^). The first stimulation parameter settings (130 Hz, 60 μs, and 2.8 V) did not reveal a significant effect (e.g., weight loss) during the first 6 months following surgery. Interesting, after a slight adjustment (50 Hz, 210 μs, and 3–4 V) the patient had a consequent weight loss (12 kg) without significant dietary changes and a reduction in food craving (Hamani et al., 2008). Because the patient reported difficulties in sleeping, the stimulation was turned off at night. The patient then began to eat at night and had increased weight (Wilent et al., 2010; Whiting et al., 2013). Wilent et al. (2010) used the same target as Hamani et al. (2008), however, in their case the stimulation (135 Hz, 60 μs, and 1–7 V) was associated with an unexpected panic event, and resulted in the interruption of the treatment.

An open-label clinical trial of hypothalamic DBS for morbid obesity is currently in progress and six patients will be included (De Salles et al., 2018). The subjects are required to have a BMI greater than 40 kg/m^2^ or to have undergone bariatric surgery with therapeutic failure, as defined by BMI ≥ 35 kg/m^2^ (must be 5 years after the procedure). Subjects must have no obesity comorbidities such as diabetes or cardiopulmonary abnormalities. The trial aims to assess the safety, identify possible side effects, and to optimize stimulation parameters in a paradigm of continuous VMH-DBS. Additionally, the study aims to determine if continuous VMH-DBS will lead to weight loss associated with changes in body composition, basal metabolism or food intake control (De Salles et al., 2018).

### Lateral Hypothalamic Area (LHA) DBS

Weight gain has been observed in patients with PD treated with the subthalamic nucleus (STN) DBS and globus pallidus internus (GPi) DBS (Novakova et al., 2007). The weight gain could be an influence of spreading electrical currents into hypothalamic pathways though this remains speculative. There are also other factors including impulse control and dyskinesia reduction which could confound the finding. Nevertheless, it is interesting that STN and GPi DBS for Parkinson’s disease have both been associated with weight gain.

A right-side LHA hypothalamotomy lesion in two patients and bilateral in one (staged over 3 months) resulted in a transient decrease in caloric intake. This finding was compared to two patients with transient stimulation but no lesion(s) (Quaade et al., 1974).

In 2013, the first DBS treatment with electrodes implanted in the LHA was performed in three patients, (two females). All patients were previously treated with gastric bypass between 2001 and 2003 (Whiting et al., 2013). During the intraoperative microelectrode recording isolated but not discernible firing patterns were recorded. Microstimulation within the LHA produced sensations of nausea and thermal responses while more ventromedial microstimulation (presumably within the VMH) resulted in an anxiety or panic response. DBS was initiated 2 months after surgery. No significant weight loss trends were observed when DBS was programmed utilizing standard settings similar to the high-frequency parameters used for movement disorder surgery. Weight loss trends were observed when monopolar DBS stimulation was applied via specific contacts found to be associated with an increase in the resting metabolic rate as measured in a respiratory chamber. After 35 months of follow-up, two patients lost weight and one had a decrease in binge eating behaviors. There were no significant neuropsychological changes across patients and no significant side effects were reported.

Hyperphagia in Prader–Willi syndrome (PWS) is an important cause of genetic obesity. Recently, Talakoub et al. (2017) recorded the local field potentials (LFPs) from the DBS contacts implanted in the LHA of a 19-year-old Prader–Willi male patient undergoing DBS for obesity. During hunger exposure to food-related cues, there was an induced increase in beta/low-gamma activity. During satiety, the recordings were marked by alpha rhythms. An alpha frequency DBS was delivered prior to and during food intake. The patient reported a sensation of fullness but had a persistent food craving. The long-term effects have yet to be reported (Talakoub et al., 2017).

Franco et al. (2018) described four patients (two males) with Prader–Willi syndrome, treated with lateral hypothalamus DBS. The cohorts age ranged from 18 to 28 and their mean (SD) baseline BMI was 39.6 (11.1). Two patients had previous bariatric surgery. All patients had psychiatric comorbidities, including skin picking, nail biting, aggressive behavior, hypersexuality, episodes of hypomania, psychosis, and impulsiveness. These behaviors were reported as adequately controlled with medications. After DBS implantation, the treatment included the following phases: titration (1–2 months), stimulation off (2 months), low-frequency DBS (40 Hz; 1 month), washout (15 days), high-frequency DBS (130 Hz; 1 month), and long-term follow-up (6 months). Six months after receiving DBS at the “best” settings, the patients had a mean 9.6% increase in weight, a 5.8% increase in BMI, an 8.4% increase in abdominal circumference, a 4.2% increase in neck circumference, a 5.3% increase in the percentage of body fat, and a 0% change in calorimetry (as compared to baseline). The hormonal levels and results of a blood workup, sleep studies, and neuropsychological evaluations were unchanged by DBS therapy. Two patients developed manic symptoms during the titration phase, one improved with DBS programming and one required an increase in topiramate. One patient was receiving preoperative testosterone injections for hypogonadism, which infrequently resulted in priapism. One episode of priapism during the titration phase required drainage. One patient developed an infection over the connector site thought to be associated with skin picking 7 months following treatment. The family requested explantation since the device was ineffective for obesity. Another patient developed a superficial infection over IPG 1 month after the implant and this issue was successfully addressed with antibiotics.

## Nucleus Accumbens DBS

The first clinical report on NA DBS was published by Mantione et al. (2010) who treated refractory obsessive-compulsive disorder with associated nicotine dependence and also obesity (Mantione et al., 2010). In this study, a 47-year-old female patient weighed 107 kg with a height of 170 cm, corresponding to a BMI of 37 was implanted with a monopolar DBS (185 Hz, 90 μs, and 3.5 V). The clinical team utilized the two deepest contacts (0 and 1) for initial programming (accumbens region) and adjusted it using the more dorsal 1 and 2 contacts 3 weeks later, when she gradually started to improve her obsessive-compulsive symptoms. Within 5 months the Yale-Brown Obsessive-Compulsive Scale scores decreased from 38 to 2 and she reported reducing the time spent on obsessions and compulsions from 20 h a day to less than 1 h a day. The HAM-A (subclinical anxiety) and the HAM-D (subclinical depression) gradually decreased from 17 to 2 and 11 to 3, respectively. Seven months after surgery she realized she was no longer dependent on her compulsions, but that she was still dependent on her cigarettes. She stopped smoking and stated she did not crave cigarettes and did not experience withdrawal symptoms. The 2-year follow-up evaluation showed her weight was 71 kg, she was not smoking and she had no desire to start smoking. She scored 1 on the Yale-Brown Obsessive-Compulsive Scale, 3 on the HAM-A, and 3 on the HAM-D.

More recently in 2016, Harat et al. (2016) described a 19-year-old woman with obesity associated with a hypothalamic region that was damaged by a craniopharyngioma (neurosurgery was in 2004). She was approved for bariatric surgery but preferred DBS surgery. She was treated with bilateral NA DBS (130 Hz, 208 μs, and 2–3.75 mA). After 14 months her weight was reduced from 151.4 kg to 138 kg and her BMI from 53 to 48. No neuropsychological or other side effects were observed as a result of the surgery (Harat et al., 2016).

Tronnier et al. (2018) reported that bilateral NA DBS (130 Hz, 90 μs, and 4 V) decreased body weight, BMI and binge behavior without significant psychological impairment in a woman who was followed for 14-months. She had depression resistant to electroconvulsive therapy (ECT). Her peak weight in November 2013 was 183.6 kg which at a height of 167 cm translated to a BMI of 66. In March 2015, she underwent gastric bypass surgery after a course of behavioral therapy for her obesity and it was reported to have limited success. Because the temporary improvement of depression was less than 2 weeks and further antidepressant medication was not successful, the decision was made for treatment with NA DBS in November 2015. Morbid obesity of the patient was considered a secondary target of the DBS procedure. After DBS surgery, her weight loss accelerated to 2.85 kg/month. At the time of writing of this report (12/2016) her body weight has continuously decreased to 106 kg, corresponding to a BMI of 38. Her depressive symptoms have also been reported to be significantly reduced. Interestingly, the weight loss was accompanied with heightened feelings of “self-efficacy of ingestive behavior” that was not felt following bariatric surgery. This case raised the issue as to whether obesity associated with depression could be addressed with NA DBS.

Rezai et al. (2018) described their experience with NA DBS for obesity in three female patients followed by their multidisciplinary team. The inclusion criteria were age between 22 and 60 years, “extreme obesity” defined as BMI > 40, and patients had to fail Rou-en-Y gastric bypass surgery. Although all patients lost weight with DBS, only one participant successfully completed the 3-year trial, losing 100 pounds with a 30% BMI reduction (55.7–39.3). After 13 months in the study, one subject requested explantation. One patient died as a result of a completed suicide after 27 months into the study. All patients had significant psychiatric illness and during the trial reported major psychosocial stressors including family violence, divorce, job loss, family illness, and pet death. The study team concluded that DBS itself was not responsible for the suicide, patient withdrawal or explantation. Regarding the association between obesity and suicide, a recent systematic review and meta-analysis of 15 prospective studies showed an inverse association between obesity with suicide mortality and attempted suicide (Amiri and Behnezhad, 2018). The mechanisms related to an association between DBS and suicide are not understood but the recent Veterans Affairs study with a long term follow-up did not show an association with STN and GPi DBS and suicide (Weintraub et al., 2013). One confounding factor has been the early reports of suicide led most major groups to implement more careful pre-operative screening and post-operative monitoring.

Rezai et al. (2018) has suggested the following strategies to reduce suicide risk (i) Requirement of close psychiatric monitoring by clinicians trained in bariatric psychiatry and mandatory psychological therapy during the study period; (ii) Requirement of subject commitment to compliance with the study requirements and close involvement with a social support system and a study partner (a family member or close friend committed to the trial with the subject) from the subject’s environment; (iii) Upfront consideration should be given to the long-term follow-up of the DBS system after the trial conclusion and should be discussed with the subjects and family support system in advance of implantation.

Finally, because of its assumed role in reward-related behavior, the ventral anterior limb of the internal capsule (vALIC) could be a potential target for obesity DBS. However, Linssen et al. (2017) reported no significant change in body weight on a group level after vALIC DBS (mean follow-up was 3.8 years, range 10 months to 8.7 years) for either obsessive-compulsive disorder (*n* = 30) or major depressive disorder (*n* = 16). The average baseline BMI in their sample of 46 patients was 28.0 (SD 7.3), with 26 (57%) being overweight (BMI 25–30, *n* = 11), obese (BMI 30–40, *n* = 12), or morbidly obese (BMI ≥ 40, *n* = 3).

## Conclusion and Final Remarks

Prevention of obesity is a worldwide issue. It will required broad changes in the worldwide pattern of food ingestion and physical activity. The cost-effectiveness of innovations must be taken into account in epidemiological terms. However, in selected cases of morbid obesity, DBS, similar to gastric surgery, may in the future be refined into a therapeutic modality. Hypothalamic DBS for obesity has been shown to be reasonably safe in well-selected patients. The effectiveness has, however, not been shown to be robust or reproducible. Based on both biological plausibility and on observational studies, the NA has emerged as an alternative obesity DBS target.

Future studies should also focus on better understanding the patient characteristics most likely to benefit from obesity DBS (Tronnier et al., 2018). Specific DBS targets may be optimal for specific clinical phenotypes. The therapy could thus become more personalized (Famm et al., 2013).

The identification of electrical control signals may provide an opportunity for closed-loop adaptive DBS systems to address obesity (Carron et al., 2013; Kuhn and Volkmann, 2017). Metabolic and hormonal sensors such as glycemic levels (Christiansen et al., 2018), leptin and ghrelin levels are candidate control signals for DBS.

Newer approaches for obesity DBS should be explored. Focused excitation or alternatively inhibition of regions of the hypothalamus may provide better outcomes compared to non-selective DBS. Utilization of the delta oscillation (Halpern et al., 2011) or other physiological markers from one or multiple regions in the obesity network is a promising approach.

Finally, it will be important to implement expert multidisciplinary screening teams as well as post-operative monitoring to lessen the occurrence of neuropsychiatric adverse events.

## Author Contributions

DF, JG, HM, TE, RZ, AL, and RW wrote the manuscript. TE, JG, and DF elaborated the tables and figures. AL, JG, MO, and RW participated in the conception of the idea and revised the manuscript.

## Conflict of Interest Statement

The authors declare that the research was conducted in the absence of any commercial or financial relationships that could be construed as a potential conflict of interest.
